# A novel *Glycyrrhiza glabra* extract liquiritin targeting NFATc1 activity and ROS levels to counteract ovariectomy-induced osteoporosis and bone loss in murine model

**DOI:** 10.3389/fphar.2023.1287827

**Published:** 2023-11-08

**Authors:** Guoju Hong, Lin Zhou, Guanqiang Zheng, Xiaoxia Zheng, Zhenqiu Chen, Wei He, Qiushi Wei

**Affiliations:** ^1^ Traumatology and Orthopedics Institute, Guangzhou University of Chinese Medicine, Guangzhou, Guangdong, China; ^2^ Department of Orthopaedics, The Third Affiliated Hospital of Guangzhou University of Chinese Medicine, Guangzhou, Guangdong, China; ^3^ Key Laboratory of Biological Targeting Diagnosis, Department of Endocrinology, Therapy and Rehabilitation of Guangdong Higher Education Institutes, The Fifth Affiliated Hospital of Guangzhou Medical University, Guangzhou Medical University, Guangzhou, China; ^4^ Department of Rehabilitation, The Third Affiliated Hospital of Guangzhou University of Chinese Medicine, Guangzhou, Guangdong, China; ^5^ The Third Clinical Medical College, Guangzhou University of Chinese Medicine, Guangzhou, Guangdong, China; ^6^ Department of Orthopaedics, The First Affiliated Hospital of Guangzhou University of Chinese Medicine, Guangzhou, Guangdong, China

**Keywords:** liquiritin, osteoclast, NFAcT1, RANKL, osteoporosis

## Abstract

Osteoporosis, a prevalent osteolytic condition worldwide, necessitates effective strategies to inhibit excessive bone resorption by curbing osteoclast hyperactivation. Liquiritin (LIQ), an flavanone derivative employed in acute lung injury and rheumatoid arthritis treatment, possesses an unclear role in addressing excessive bone resorption. In this investigation, we found that LIQ demonstrates the ability to inhibit osteoclast formation and the bone-resorbing activity induced by RANKL. At a specific concentration, LIQ significantly attenuated NF-κB-Luc activity induced by RANKL and curtailed NF-κB activation in RANKL-stimulated RAW264.7 cells, resulting in reduced IκB-α breakdown and diminished nuclear NF-κB levels. Furthermore, LIQ markedly inhibited RANKL-induced NFATc1 activation, as evidenced by diminished NFATc1 luciferase activity, reduced NFATc1 mRNA levels, and decreased nuclear NFATc1 protein levels. Subsequent experiments demonstrated that LIQ effectively restrained the RANKL-induced elevation of intracellular calcium as well as reactive oxygen species. Additionally, LIQ exhibited a downregulating effect on the expression of osteoclast-specific genes, which include *Acp5*, *Cathepsin K*, *Atp6v0d2*, *Nfatc1*, *c-Fos*, and *Mmp9*. Notably, our findings revealed the potential of LIQ to counteract decreased bone density in mice that underwent ovariectomy. Collectively, the data indicate that LIQ impedes osteoclast formation triggered by RANKL and the subsequent reduction in bone mass by mitigating ROS levels and suppressing the Ca^2+^/MAPK-NFATc1 signaling pathway, suggesting its promising candidacy as a therapeutic agent for RANKL-mediated osteoporosis.

## Introduction

Bones, as dynamic organs, undergo constant remodeling through the coordinated actions of osteoblasts, responsible for bone formation, and osteoclasts, which mediate bone resorption (Katsimbri, 2017; Majidinia et al., 2018). Among age-related conditions, osteoporosis is particularly prevalent in postmenopausal women and is characterized by an imbalance between osteoclast and osteoblast activity (Hart et al., 2020; Author Anonymous, 2021). Enhanced osteoclast function leads to excessive bone resorption, compromising the overall structural integrity of the skeleton (Kim et al., 2020). Consequently, the identification of agents capable of suppressing osteoclastogenesis and bone resorption holds promise for the prevention and treatment of pathological bone loss (Reid and Billington, 2022). Natural compounds present an intriguing avenue for exploring potential therapeutic options for osteoporosis in the elderly (Xu et al., 2022). Therefore, conducting screenings to identify natural compounds with anti-osteoclastic effects is of great interest.

Osteoclasts, derived from the monocyte-macrophage lineage, fulfill essential roles in both physiological development and various pathological conditions (Kodama and Kaito, 2020). The regulation of osteoclast differentiation is intricately controlled by a multitude of cytokines and signaling pathways. Notably, two pivotal elements, specifically macrophage colony-stimulating factor (M-CSF) and receptor activator of nuclear factor kappa-B ligand (RANKL), exert significant control over the differentiation of osteoclasts in both laboratory settings and within living organisms (Udagawa et al., 2021). M-CSF facilitates the multiplication and continued existence of precursor cells that develop into osteoclasts while upregulating the expression of RANK, a crucial prerequisite for initiating osteoclast differentiation (Gong et al., 2022). After RANKL binds to RANK, it triggers the recruitment of tumor necrosis factor receptor-related factors (TRAFs) 2, 3, 5, and 6, thereby activating the mitogen-activated protein kinase (MAPK) and nuclear factor kappa-B (NF-κB) pathways (Yasuda, 2021). Early-phase NF-κB activation is noticeable, along with the breakdown of its subunits, p50 and p52, contributes to osteoclast dysgenesis and the development of an osteoporotic phenotype (Yao et al., 2021). Moreover, there are heightened levels of reactive oxygen species (ROS) within osteoclasts which has been implicated in promoting both osteoclast formation and activation. Importantly, heightened ROS production has been associated with pathological bone resorption linked to estrogen deficiency and inflammatory arthritis (Agidigbi and Kim, 2019; Zhu et al., 2022).

The activation of these signaling pathways facilitates the translocation and subsequent activation of NFATc1, a pivotal transcription factor crucial for osteoclastogenesis (Takayanagi, 2021). NFATc1 exerts direct control over numerous osteoclast-associated genes, including *TRAcP*, *CTR*, and *MMP-9* (Kurotaki et al., 2020). Research has indicated that NFATc1 plays a crucial role in the development of tissues, as evidenced by the rescue of fetal lethal *Nfactc1* knockout mice upon NFATc1 expression specifically in the heart (de la Pompa et al., 1998). Remarkably, these rescued mice exhibited severe osteoporosis at birth, underscoring the critical involvement of NFATc1 in bone homeostasis (de la Pompa et al., 1998; Gu et al., 2020). Loss of *Nfactc1* in mice leads to the development of severe osteopetrosis characterized by elevated bone density and the incapacity to break down primary spongiosa, leading to the buildup of calcified cartilage (Aliprantis et al., 2008). Therefore, modulating NFATc1 expression through targeting the RANKL-induced signaling pathways holds therapeutic potential for managing osteoclast-related disorders, including postmenopausal osteoporosis.

Liquiritin (LIQ) is a naturally occurring compound derived from the root of *Glycyrrhiza glabra* (Sharifi-Rad et al., 2021). LIQ has garnered considerable scientific attention due to its potential therapeutic properties such as anti-inflammatory, antioxidant, skin-brightening, and anticancer attributes, as well as its hepatoprotective and neuroprotective effects (Qin et al., 2022). Notably, LIQ has been shown to facilitate cellular proliferation and reduce apoptosis, which has been associated with its capacity to impede oxidative stress and endoplasmic reticulum stress, thereby preserving the integrity of the blood-brain barrier (Li M. et al., 2021). Additionally, LIQ has demonstrated safeguarding properties against oxygen and glucose deprivation/reoxygenation-induced damage by enhancing cell viability, reducing apoptosis, and mitigating oxidative stress through activation of the p38 MAPK/NF-κB pathway (Liao and Zhang, 2022). In the context of osteoporosis, characterized by imbalanced bone remodeling and increased bone resorption, LIQ’s diverse pharmacological effects become particularly relevant. Its anti-inflammatory and antioxidant properties may mitigate the inflammatory microenvironment associated with osteoporosis, potentially reducing osteoclast activation and bone resorption. Indeed, given the aforementioned characteristics and the molecular mechanism associated with osteoclasts, we put forth the hypothesis that LIQ can influence the differentiation of osteoclasts by influencing the p38/MAPK, NF-κB, and ROS-related signaling pathways.

## Materials and methods

### LIQ–RANKL molecular docking

LIQ’s 2D configurations were sourced from PubChem (CID: 503737, illustrated in Supplementary Figure S1). Meanwhile, the 3D arrangements underwent transformation and ligand optimization via LigPrep from the Schrödinger Discovery Suite. Multiple potential ionization states were generated and all these variations were included in the subsequent docking step. The crystalline structure of the RANKL-RANK complex was retrieved from the PDB database and underwent processing. The initial and improved structures were compared using the Ramachandran Plot. The enhanced RANKL structure was explored with Sitemap to pinpoint binding sites. Subsequently, a receptor grid was generated to encompass all conceivable binding locations for the docking step. Throughout the docking process, the docking orientation of LIQ onto RANKL was achieved via Glide XP docking, followed by a comparison of docking scores; the orientation with the most favorable conformation was chosen. Additionally, the influence of LIQ’s bonding on protein stability and bond formation was assessed.

### *In vitro* drug screening

To generate RANKL-induced osteoclasts, extracted bone marrow macrophages (BMMs) were collected from fetal 8-week-old C57BL/6J mice. BMMs were then plated onto 96-well plates (6 × 10^3^ cells per well) with complete Alpha Modified Eagle Medium ( α -MEM, Sigma-Aldrich, St. Louis, MO, USA) containing M-CSF (50 ng/mL, R&D Systems, Minneapolis, MN, USA) to adhere. The next day, osteoclasts were cultured with medium supplemented with recombinant murine sRANK-Ligand protein (rm-sRANKL, 50 ng/mL, PeproTech, Rocky Hill, NJ, USA) with or without LIQ (0, 0.01, 0.05, 0.1, 0.2 mM), which was refreshed every 2 days. The cells gradually showed an osteoclast-like appearance, and mature osteoclasts were formed after about 5 days. Then the cells were fixed in 4% paraformaldehyde (PFA) and stained using a TRAcP kit (Solarbio Science and Technology Co., Ltd., Beijing, China). TRAcP^+^ multinucleate cells (stained nuclei > three) were counted as osteoclasts. LIQ was purchased from CSNpharm (Catalog No. CSN11310, Shanghai, P.C. China) dissolve in dimethylsulfoxide (DMSO).

### Cytotoxicity assay

The cytotoxic effects of LIQ were evaluated using an MTS assay kit, following the manufacturer’s instructions. Briefly, BMMs were seeded into a 96-well plate (6 × 10^3^ cells/well), followed by a 24-h incubation. Subsequently, different concentrations (0, 0.01, 0.05, 0.1, 0.2, 0.25–1 mM) of LIQ were added to the cells for 2 days. Then, 20 μ M MTS (3-(4,5-dimethylthiazol-2-yl)-5-(3-carboxymethoxyphenyl)-2-(4-sulfophenyl)-2H-tetrazolium) solution (Promega Corporation, Madison, WI, USA) was added to the cells. The absorbance at 490 nm was measured with a microplate reader (BMG Labtech, Ortenberg, Germany).

### Hydroxyapatite resorption assay

To investigate the effects of LIQ on bone resorption of mature osteoclast, a hydroxyapatite assay was performed. BMMs (1 × 10^5^ cells per well) were seeded onto plates coated with collagen (Thermo Fisher Scientific, Waltham, MA, United States) and stimulated with rm-sRANKL and M-CSF. After BMMs had developed into mature osteoclasts, cells were dissociated and seeded into 96-well Corning^®^ osteo assay plates (Corning Inc., Corning, NY, United States) with an equal number of cells per well. Mature osteoclasts were exposed to specified concentrations of LIQ (0, 0.05, and 0.1 mM) and incubated in medium containing rm-sRANKL and M-CSF for 48 h. Half of the osteoclasts were processed with TRAcP staining as above, while the other half were bleached and removed for measuring the resorbed area. The resorbed area was photographed by microscopy and analyzed by ImageJ.

### Generation of intracellular ROS

After treating BMMs with RANKL and LIQ (0.05 and 0.1 mM) for 60 min, we cultured them in Hank’s balanced salt solution supplemented with 5 mM H_2_DCFDA (Thermo Fisher Scientific, Waltham, MA, United States). in the dark for 5 min. H_2_DCFDA, a nonfluorescent probe, undergoes oxidation and cleavage of the acetate groups, resulting in the formation of the highly fluorescent dichlorofluorescein (DCF). To detect DCF fluorescence, we employed an A1Si confocal microscope equipped with a NIKON 488 nm excitation light and 540 nm emission light.

### Calcium (Ca^2+^) oscillation measurements

BMMs were seeded into 48-well plates at a density of 1×10^4^ cells per well and cultured for 1 day. The following day, cells in the treatment group were exposed to LIQ (0.1 mM) and incubated with rm-sRANKL overnight. For the assessment of free Ca^2+^ levels in RANKL-induced osteoclasts, the cells were first washed using a washing buffer composed of Hank’s balanced salt solution containing 1 mM probenecid and 1% FBS. Subsequently, they were loaded with 100 μL/well of a Fluo4 staining solution (prepared by diluting Fluo4-AM from Thermo Fisher Scientific in Pluronic-F127 in DMSO) and incubated for 45 min at 37°C. After staining, the cells were washed again with an assay buffer. The levels of intracellular free Ca^2+^ were visualized using an inverted fluorescence microscope (Nikon, Tokyo, Japan) with an excitation wavelength of 488 nm. Images were captured every 2 s over a duration of 1 min. Cells exhibiting more than two peaks of fluorescence intensity during the observed timeframe were categorized as oscillating cells. The intensity of oscillation was quantified by calculating the average peak height per cell.

### Luciferase reporter assays of NF- κ B and NFATc1

The transcriptional status of NF-κB and NFATc1 was gauged through a luciferase reporter assay system. In brief, RAW264.7 cells obtained from the American Type Culture Collection (Manassas, VA, United States) were transfected with either p-NF-B-TA-Luc or p-NFATc1-TA-Luc luciferase reporter constructs, which are responsive to NF-κB and Nfatc1, respectively. These transfected cells were subsequently placed onto plates (1.5×10^3^ cells/well) and allowed to settle overnight. The cells were pre-treated with varying concentrations of LIQ for 1 hour. Following this, the Luc-NF-κB cells that had been pre-treated were stimulated with 50 ng/mL of recombinant mouse-sRANKL for 6 h. Meanwhile, the Luc-NFATc1 cells were treated with LIQ for 24 h. Subsequent to these treatments, the cells were lysed using a luciferase reporter assay kit from Promega Corporation (Madison, WI, United States), and the luminescent signal produced by luciferase activity was quantified.

### Quantitative real-time polymerase chain reaction (qRT-PCR)

qRT-PCR was employed to assess the expression levels of several genes (*Acp5*, *Cathepsin K*, *Atp6v0d2*, *Nfatc1*, *c-Fos*, and *Mmp9*) during the process of osteoclast differentiation induced by RANKL. BMMs were seeded into six-well plates at a density of 1×10^5^ cells per well. They were then cultured in α-MEM supplemented with M-CSF and rm-sRANKL. This culture was carried out in the presence or absence of LIQ at concentrations of 0, 0.05, and 0.1 mM over a span of 5 days. Subsequent to the incubation period, total RNA was extracted from the pre-treated cells using TRIZOL^®^ reagent sourced from Life Technologies (Carlsbad, CA, United States). Following RNA extraction, 1 μg of the extracted RNA underwent reverse transcription into single-stranded complementary DNA (cDNA). This step was facilitated by Moloney murine leukemia virus reverse transcriptase (M-MLV-RT) and an oligo-dT primer from Promega (Madison, WI, United States). For the actual qRT-PCR analysis, a Real-time PCR machine manufactured by Applied Biosystems (Warrington, Cheshire, United Kingdom) was employed. The expression levels of the target genes were quantified and normalized to *Gapdh* as the reference gene. For reference, the specific primer sequences utilized in the qRT-PCR analysis are available in Supplementary Table S1.

### Western blotting

For the assessment of osteoclastic protein levels within the NFATc1 and NF-κB pathways, we initiated the experiment by seeding BMMs at a density of 1×10^5^ cells per well into six-well plates. These cells were cultivated in a medium supplemented with rm-sRANKL and M-CSF. Importantly, this was performed in the presence or absence of LIQ at a concentration of 0.1 mM. During the GTP-Rac1 assessment, the cell lysis solution underwent incubation with the PAK1 PBD protein sourced from Sigma-Aldrich (St. Louis, MO, United States). Subsequently, we subjected the samples to analysis using the active Rac1 PullDown and Detection Kit obtained from Cell Signaling Technology (Danvers, MA, United States). For protein extraction, cells were lysed using radioimmunoprecipitation assay (RIPA) lysis buffer procured from Millipore (Burlington, MA, United States). From these lysates, total protein was isolated. Protein concentrations were determined utilizing the Bradford method in accordance with the manufacturer’s instructions from Bio-rad (Hercules, CA, United States). Subsequently, equal quantities of cellular protein were subjected to separation through sodium dodecyl sulfate (SDS)-polyacrylamide gel electrophoresis. The separated proteins were then transferred onto a polyvinylidene fluoride (PVDF) membrane provided by GE healthcare (Chicago, IL, United States). Following transfer, membranes were subjected to blocking using 5% skim milk within a TBS-T buffer. These blocked membranes were then incubated overnight at 4°C with the respective primary antibodies. After this, secondary antibodies were applied and incubated for 2 h. The detection of protein bands occurred through visualizing the blots using an Image-quant LAS 4000 system from GE Healthcare (Chicago, IL, United States).

### Murine ovariectomy (OVX)-induced osteoporosis model

Following the Institutional Animal Ethics Committee’s approved guidelines, animal experiments were conducted. A cohort of 36 female C57BL/6J mice was individually housed in ventilated cages under specific pathogen-free conditions. The mice were allocated into three groups through random assignment: the sham-operated group (*n* = 12), the OVX group (*n* = 12), and the OVX + LIQ group (*n* = 12). After a 1-week acclimatization, mice were anesthetized and underwent bilateral OVX surgery for the OVX and OVX + LIQ groups, while the sham group underwent a sham procedure (Hong et al., 2021). For the OVX + LIQ group, mice were intraperitoneally injected with 20 mg/kg of LIQ (dissolved in DMSO) every other day for 6 weeks. Prior to the formal experiment, a pre-experiment was conducted to determine the appropriate and effective dose of LIQ for our study. During this pre-experiment, we systematically varied the doses (ranging from 5 to 30 mg/kg) of LIQ to identify the optimal dosage (20 mg/kg) that exhibited the desired inhibitory effects on bone resorption. a The sham and OVX groups received an equal volume of vehicle, prepared as 1% DMSO in PBS, during the same period. At the study’s conclusion, all mice were euthanized. The mice were subjected to the respective treatments promptly following OVX to assess the preventive or ameliorative effects of LIQ in the development of osteoporosis induced by ovariectomy.

Half of the mice from each group were utilized to assess bone homeostasis post LIQ administration. Blood samples were collected from the abdominal aortas, then centrifuged to obtain serum, and analyzed for TRAcP and C-terminal telopeptide (CTX-1) levels using R&D Systems’ enzyme-linked immunosorbent assay (ELISA) kits. The left femur were utilized for micro-computed tomography (Micro-CT) scanning to assess microstructural changes and three-point bending tests. Histomorphology analysis was performed on the right femur. The remaining half of the mice underwent *in vivo* biosafety testing for LIQ. Organ samples were extracted for evaluation of liver, spleen, lung, heart, and renal toxicity based on organ size and surface appearance. Blood samples were collected from the aortas for complete blood count analysis using a BC6800 automated analyzer.

### Micro-CT scanning

The femur samples were immersed in a 4% paraformaldehyde solution for a 24-h period to facilitate fixation. Following fixation, a specific region of interest (ROI) located within the trabecular bone, situated 0.5 mm away from the femur’s growth plate, was subjected to analysis using a high-resolution Micro-CT system provided by Scanco Medical (Wangen-Brüttisellen, Switzerland). The subsequent quantitative analysis focused on various trabecular parameters, encompassing bone volume per tissue volume (BV/TV), trabecular thickness (Tb.Th.), trabecular number (Tb.N), and connectivity density (Conn.Dn). To present the findings, both two-dimensional and three-dimensional images were generated employing DataViewer and CTvol software, which is sourced from Bruker micro-CT (Kontich, Belgium).

### Histomorphometric analysis

Subsequent to the Micro-CT analysis, the femur specimens underwent a meticulous collection process and were subsequently subjected to additional processing steps. Initially, the specimens were fixed and subsequently immersed in a 10% ethylenediaminetetraacetic acid (EDTA) soaking solution for an extended duration of 3 weeks. This immersion was continued until the specimens achieved a softened consistency suitable for further manipulation. Following this, the specimens were embedded into paraffin blocks to enable the subsequent processes of sectioning and staining. To facilitate sectioning, a microtome was utilized to produce histological sections, each having a thickness of 4 μm. With a focus on assessing osteoclastic activity, these sections underwent specific staining procedures, including TRAcP staining as well as hematoxylin and eosin (H&E) staining. Subsequent to the staining procedures, the sections were carefully examined using a microscope to ascertain the resultant patterns. For a quantitative evaluation of parameters related to osteoclasts, such as osteoclast surface/bone surface (Oc.S/BS) and osteoclast number/bone surface (N.Oc./BS), an in-depth analysis was conducted.

### Statistical analysis

The data presented in this study were obtained from *in vitro* and *in vivo* experiments at least conducted in triplicate, ensuring robustness and reliability. The results are expressed as mean values ±standard deviation (SD) to provide a measure of the variability within each dataset. Statistical analysis was performed by SPSS Statistics (version 26.0) using Student’s t-test to compare the means between groups, and *p*-values below 0.05 were considered statistically significant, indicating meaningful differences between the compared groups.

## Results

### Identification of affinity of LIQ-RANKL complex

**FIGURE 1:**
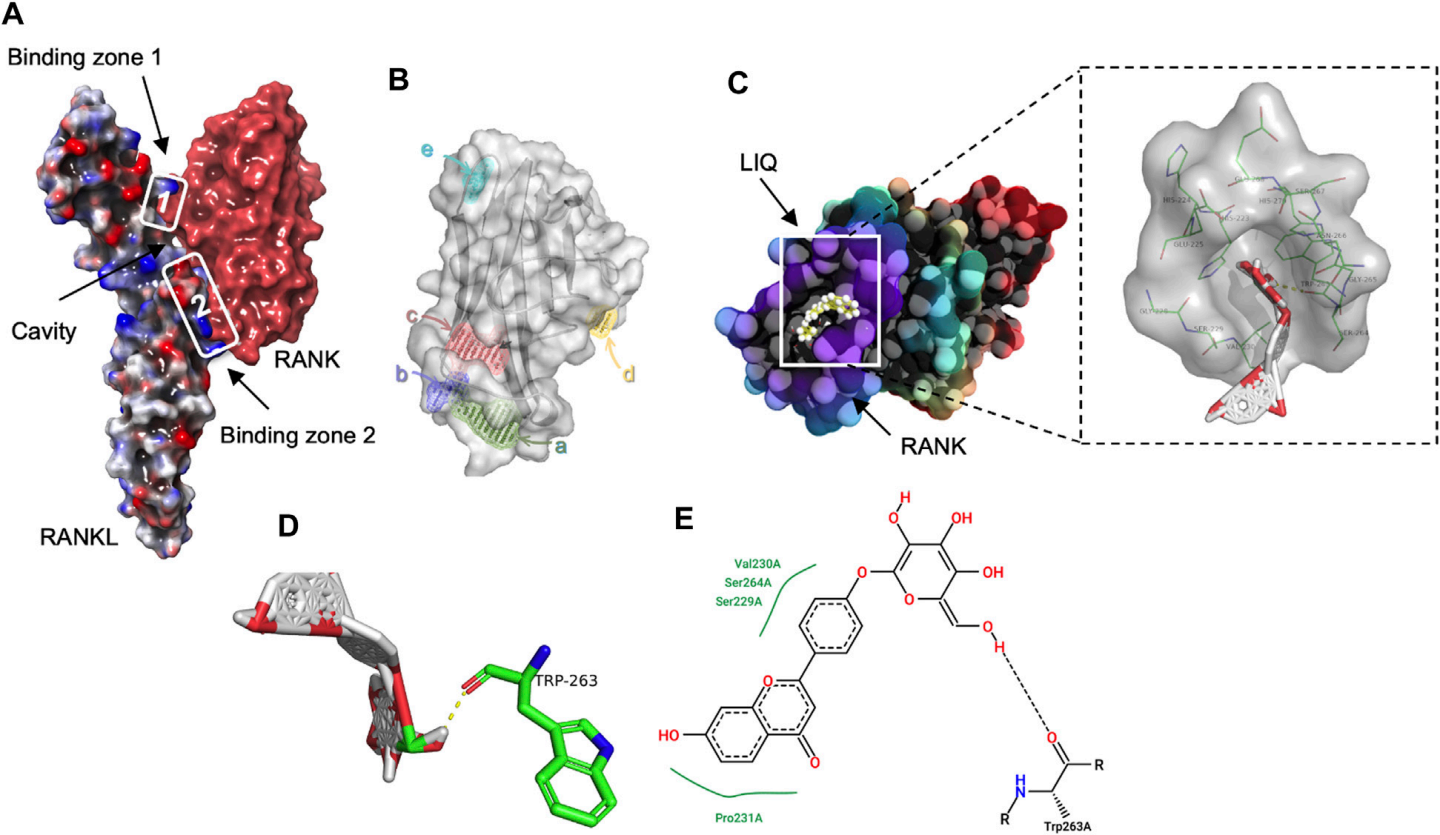
Exploring the LIQ-RANKL Interaction Through Computational Docking Analysis. **(A)** A three-dimensional (3D) depiction highlights the discontinuous nature of the RANK/RANKL complex interface, forming two distinct binding regions that generate a cavity between the cytokine and receptor. **(B)** The images portray the potential binding sites of RANKL. **(C)** Structural images showcase the docking pose and interactions between RANKL and LIQ, with amino acid residues participating in bonding highlighted in green. **(D)** The 3D structural view captures the binding interaction of LIQ with RANKL. Green highlights signify amino acid residues engaged in the binding, while yellow lines indicate hydrogen bonds established with polar atoms. **(E)** The two-dimensional structural view presents the intricate interactions between RANKL and LIQ.

For the assessment of the synergistic effects between LIQ and RANKL, a computational docking procedure was executed to ascertain the binding affinities of LIQ and RANKL. Initially, the crystal model of the RANK-RANKL protein complex was established, and a comprehensive exploration of their bioactivity center was undertaken (Figure 1A). Subsequently, through this exploration, a potential binding site for LIQ onto RANKL was pinpointed within a hydrophobic region exhibiting both hydrogen-bond acceptor and donor characteristics (Figures 1B, C). Remarkably, this identified binding site for the LIQ docking process was situated at the residues TRP263 on the RANKL molecule (Figures 1D, E). The outcome of quantitative computational docking indicated a substantial affinity between RANKL and LIQ, with a calculated binding free energy of −8.08 kcal/mol. These observations led us to conclude that the robust affinity between LIQ and RANKL originates from intricate non-covalent interactions between the two entities.

### LIQ attenuates RANKL-induced osteoclastogenesis

**FIGURE 2:**
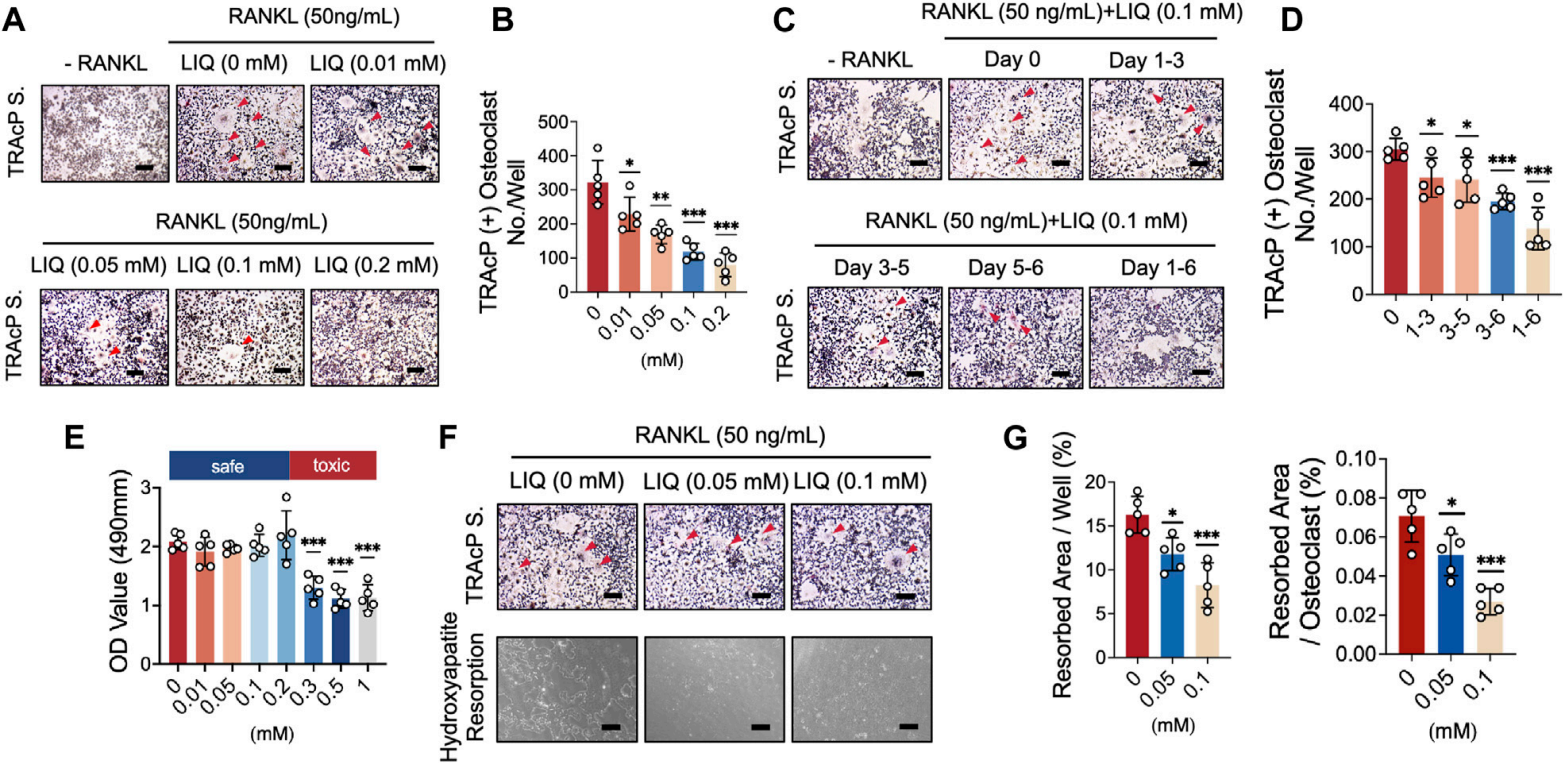
LIQ suppresses RANKL-induced osteoclastogenesis and hydroxyapatite bone resorption while preserving cell viability. **(A)** BMM cells were cultivated with M-CSF and RANKL (50 ng/mL) while exposed to varying LIQ concentrations for 5 days. TRAcP staining showcased a dose-dependent inhibition of RANKL-induced osteoclast differentiation by LIQ, displaying an IC_50_ value of 0.1 mM. Representative images are shown to illustrate this effect. Scale bar = 10 μm. **(B)** Quantitative analysis of the count of TRAcP-positive multinuclear cells (containing three or more nuclei) under different LIQ treatments (*n* = 5). **(C)** BMMs treated with LIQ for specified durations were assessed using TRAcP staining. Representative images are displayed. Scale bar = 100 μm. **(D)** Quantitative analysis of multinucleated osteoclast-like cells after exposure to LIQ for various time intervals (*n* = 5). **(E)** BMMs were treated with diverse LIQ concentrations for 48 h, and cell viability was determined via the MTS assay. **(F)** Representative images depicting hydroxyapatite resorption areas correlated with the count of TRAcP-stained osteoclasts. Scale bar = 100 μm. **(G)** Quantification of the count of TRAcP-positive multinucleated cells after LIQ treatment, alongside the percentage of hydroxyapatite surface area resorbed per osteoclast post-LIQ treatment. Statistical significance was assessed by comparison with the RANKL-treated group (*n* = 5). (*p-value <0.05, **p-value <0.01, ***p-value <0.001, *versus* RANKL-treated control).

An osteoclastogenesis assay was performed to measure the inhibitory effects of LIQ on RANKL-induced osteoclastogenesis. The TRAcP staining results demonstrated that LIQ dose-dependently suppressed the formation of TRAcP^+^ multinucleate osteoclasts (>three nuclei/cell) (Figures 2A, B). To identify which stage of osteoclastogenesis is affected, LIQ IC_50_ as 0.1 mM (Half-maximal inhibitory concentration) was added to BMMs cultured in medium containing rm-sRANKL and M-SCF at indicated time intervals. LIQ exerted its inhibitory effects predominantly from day 3–6 of osteoclast differentiation (Figures 2C, D). An MTS assay was performed to evaluate whether the effects of LIQ on osteoclastogenesis could be due to its cytotoxicity. Our results showed that LIQ concentrations up to 1 mM (Figure 2E) did not reduce BMM proliferation and hence were not cytotoxic.

### LIQ reduces bone resorption activity of mature osteoclasts

A mature hydroxyapatite absorption test was used to evaluate the effects of LIQ on the absorptive function of mature osteoclasts. BMMs were differentiated into osteoclasts, which were transferred to hydroxyapatite-coated plates, simulating bone. As shown in Figures 2F, G, LIQ (0.05, and 0.1 mM) reduced the absorptive activity of osteoclasts. We observed a small decrease in the number of mature osteoclasts at a LIQ concentration of 0.1 mM. These data indicate that in addition to reducing the differentiation of osteoclasts, LIQ also significantly inhibited the absorption of hydroxyapatite by osteoclasts.

### LIQ reduces ROS production during osteoclastogenesis

**FIGURE 3:**
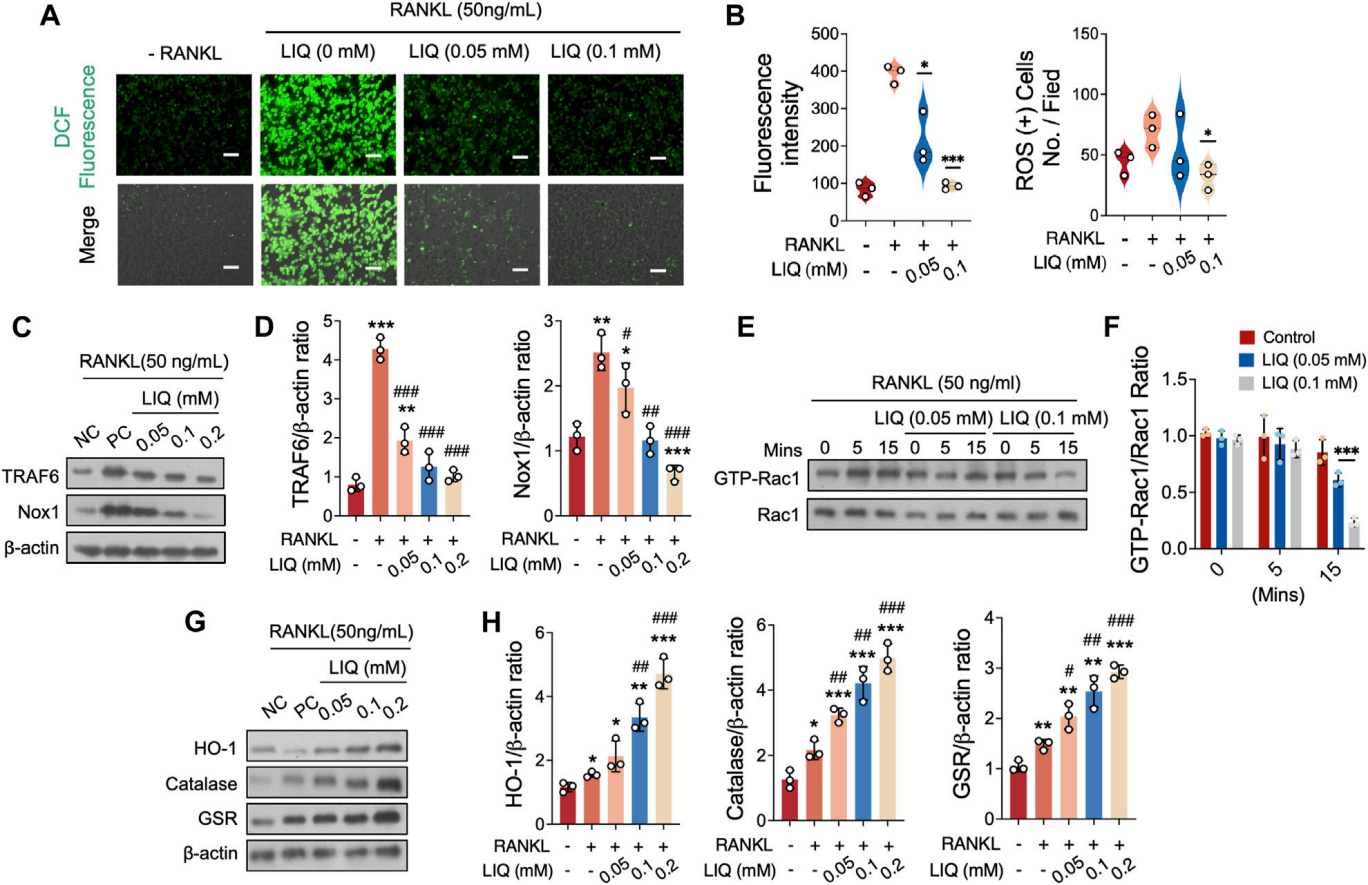
LIQ Regulation of ROS Levels and ROS Signaling Pathway in Osteoclasts. **(A)** Depictions illustrate intracellular ROS generation stimulated by RANKL within BMMs, visualized using the oxidation-sensitive dye H_2_DCFHDA. Upper panel showcases green fluorescent DCF emission, while the lower panel combines DCF fluorescence with differential interference contrast for each group. Scale bar: 100 μm. (*p-value <0.05, **p-value <0.01, ***p-value <0.001). **(B)** Comprehensive quantification of DCF fluorescence intensity per osteoclast (*n* = 3), alongside quantitative evaluation of ROS-stained cell count per field (*n* = 3). **(C)** Depicting protein expression of TRAF6 and NOX1 in RANKL-treated osteoclasts upon LIQ addition. Protein levels are normalized to β-actin. **(D)** Quantitative assessment of TRAF6 and NOX1 normalized to β-actin (*n* = 3). (*p < 0.05, **p < 0.01, ***p < 0.001, *versus* blank control. #*p* < 0.05, ##*p* < 0.01, ###*p* < 0.001, *versus* RANKL-treated control). **(E)** Representative images demonstrating GTP-Rac1 expression at different time points (0, 5, and 15 min) post LIQ treatment. Cells cultured with PAK1-PBD protein, protein expression normalized to Rac1 levels. **(F)** Quantitative analysis of GTP-Rac1 normalized to Rac1 (*n* = 3). (***p-value <0.001). **(G)** Depicting representative images of HO-1, CAT, and GSR protein expression. **(H)** Quantitative analysis of protein band signal intensity for HO-1, CAT, and GSR, normalized to β-actin (*n* = 3). (*p < 0.05, **p < 0.01, ***p < 0.001, *versus* blank control. #*p* < 0.05, ##*p* < 0.01, ###*p* < 0.001, *versus* RANKL-treated control).

We further showed that LIQ treatment inhibited the RANKL-induced upregulation of ROS levels in a dose-dependent manner without affecting the number of BMMs. In the presence of LIQ, ROS inhibited the transformation of DCFH-DA to the highly fluorescent DCF (Figures 3A, B). LIQ can effectively inhibit the RANKL-induced differentiation of osteoclasts by reducing intracellular ROS levels.

To gain insights into how LIQ regulates ROS levels, we investigated the underlying regulatory mechanism involving LIQ’s impact on NADPH oxidase 1 (NOX1) activation, the primary contributor to ROS generation. Our fingdings demonstrated that the expression of NOX1 significantly increased upon stimulation with RANKL. However, when different concentrations of LIQ (0.01, 0.05, and 0.1 mM) were administered, it effectively inhibited this elevation (Figures 3C, D). To explore the specific pathways involved, we examined the potential modulation of tumor necrosis factor receptor-associated factor 6 (TRAF6) and GTP-bound Rac1 (GTP-Rac1) levels by LIQ to assess its effect on NOX1 activity. Following RANKL treatment, we observed an upregulation of TRAF6 expression, which was subsequently downregulated upon administration of LIQ at concentrations of 0.01, 0.05, and 0.1 mM (Figures 3C, D). GTP-Rac1, an intracellular factor triggering NOX1 activation, exhibited a significant enhancement in activation after 5 min of stimulation with rm-sRANKL and then decrease after 15 min. Notably, LIQ administration in a dose-dependent manner (0.05 and 0.1 mM) effectively suppressed this activation (Figures 3E, F).

### LIQ increases the activity of antioxidant enzymes

To determine whether LIQ has the ability to decrease ROS levels by increasing the activity of antioxidant enzymes, we evaluated the expression of various enzymes, including heme oxygenase-1 (HO-1), catalase (CAT), and glutathione disulfide reductase (GSR). Our findings demonstrated that exposure to RANKL caused a partial reduction in the expression of these enzymes. However, the administration of LIQ in osteoclasts restored their expression (Figures 3G, H). These results collectively indicate that LIQ employs a dual mechanism in reducing intracellular ROS levels induced by RANKL: it inhibits ROS generation and enhances ROS scavenging by upregulating antioxidant enzymes.

### LIQ inhibits Ca^2+^ oscillations in osteoclast differentiation

**FIGURE 4:**
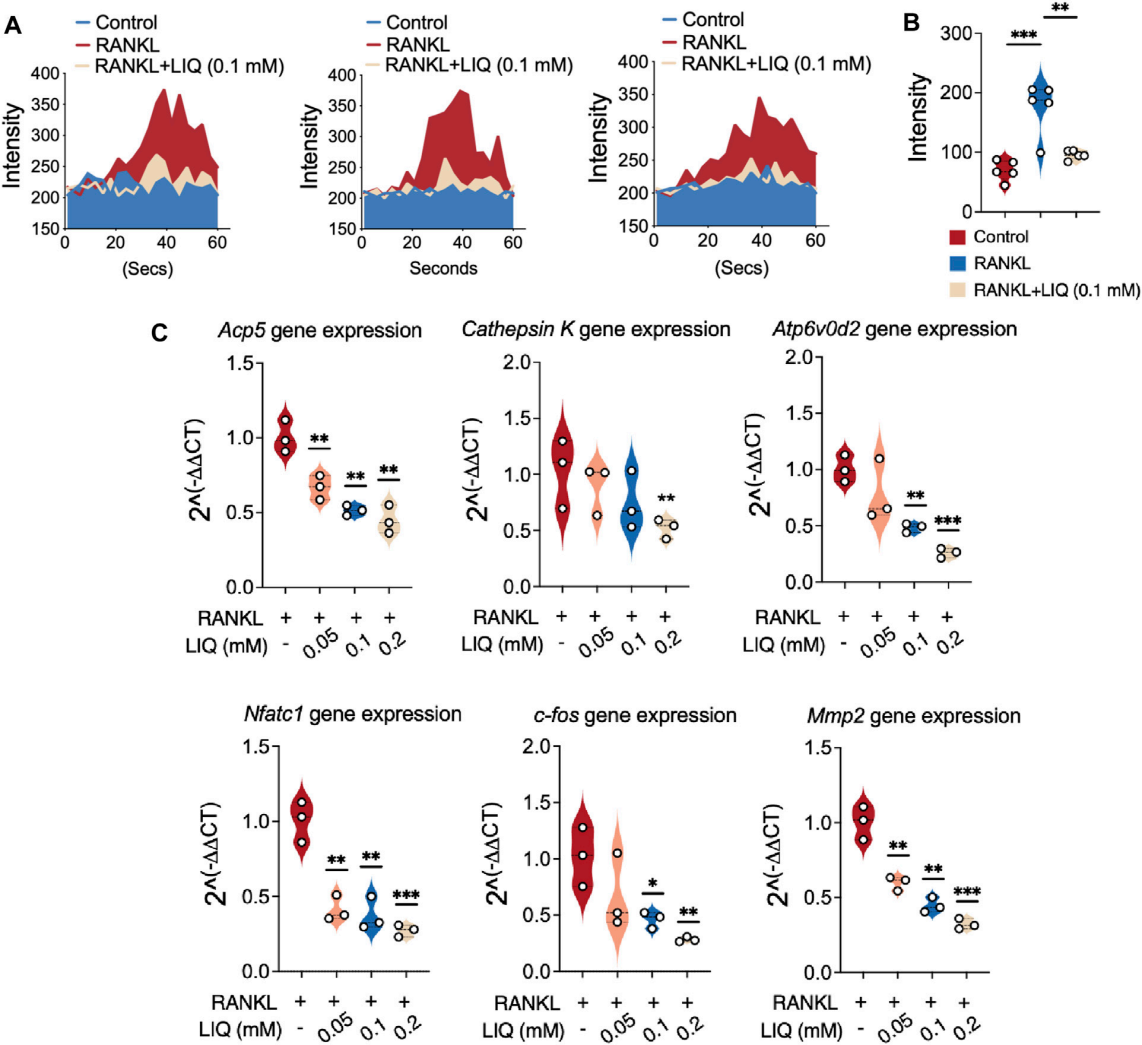
Suppression of RANKL-induced calcium oscillation and osteoclast-related gene expression in BMMs by LIQ. **(A)** Calcium dynamics were recorded in various experimental groups: the RANKL-only group, the M-CSF-treated group (sham), and the group treated with both RANKL and LIQ (0.1 mM). (*p-value <0.05, **p-value <0.01, ***p-value <0.001). **(B)** The average cellular intensity change per well was quantified. Calcium oscillations were analyzed based on cell conditions, and the difference between the maximum peak intensity and the baseline intensity was calculated. **(C)** qRT-PCR was employed to measure gene expression levels, which were subsequently normalized to *Gapdh*. The examined genes included *Acp5, Cathepsin K, Atp6v0d2*, *Nfatc1, c-Fos,* and *Mmp9*. (*p-value <0.05, **p-value <0.01, ***p-value <0.001, *versus* RANKL-treated control).

The oscillations of Ca^2+^ induced by RANKL play a pivotal role in stimulating signal transduction pathways that subsequently lead to the activation of NFATc1. Consequently, our investigation centered on the impact of LIQ on these Ca^2+^ oscillations. As anticipated, the presence of LIQ at a concentration of 0.1 mM resulted in a noteworthy reduction of nearly 50% in RANKL-induced Ca^2+^ oscillations (Figures 4A, B). These outcomes harmoniously point toward the strong inhibitory influence exerted by LIQ on NFATc1 activation. Notably, this inhibition might be attributed to the concurrent interference with both RANKL-mediated NF-κB activation and the Ca^2+^ oscillations, which collectively contribute to the overall regulatory mechanism.

### LIQ dowregulated the expression of osteoclast marker genes

We also studied the effects of LIQ on the mRNA expression levels of osteoclast-specific genes in BMMs and newly developed osteoclasts. We found that LIQ treatment (0.1 mM) reduced the mRNA levels of the osteoclast-related genes in RANKL-induced osteoclast formation (Figure 4C). The findings unequivocally establish that LIQ exerts a strong inhibitory effect on the expression of osteoclast marker genes in an *in vitro* setting.

### LIQ inhibits RANKL-induced NF-κB activation, IκB-α degradation, ERK phosphorylation and NFATc1 signaling

**FIGURE 5:**
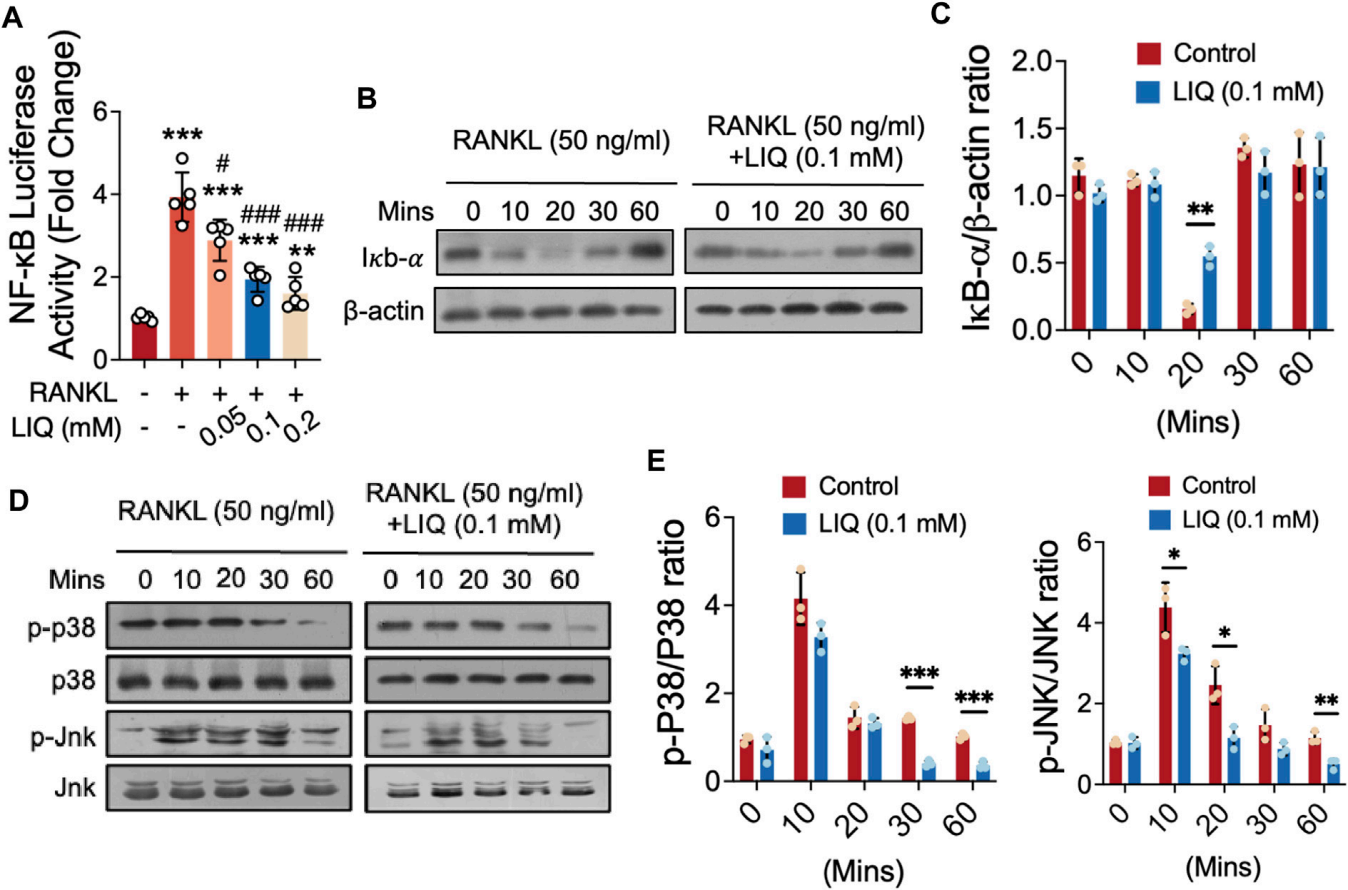
Impact of LIQ on NF-κB Activity, IκB-α Expression, and MAPK Signaling. **(A)** Measurement of luciferase activity in NF-κB-transfected RAW264.7 cells, stimulated with rm-sRANKL (50 ng/mL) following LIQ treatment. (**p-value < 0.01, ***p-value < 0.001, versus blank control. #*p*-value < 0.05, ##*p*-value < 0.01, ###*p*-value < 0.001, versus RANKL-treated control). **(B)** Protein lysates from LIQ (0.1 mM) pre-treated BMM-induced osteoclasts, followed by rm-sRANKL (50 ng/mL) stimulation at various time intervals. Western blot analysis with specific antibodies for IκB-α to β-actin was conducted. **(C)** Relative protein levels quantified by IκB-α to β-actin ratio determination. **(D)** Western blot analysis with specific antibodies for p38 and Jnk was conducted. **(E)** Quantitative analysis of p-p38 to p38 and p-Jnk to Jnk ratios using ImageJ software. (*p-value < 0.05, **p-value < 0.01, ***p-value < 0.01 compared to RANKL-treated and LIQ-untreated controls).

We investigated the effects of LIQ on RANKL-induced signal transduction by NF-κB luciferase reporter gene assay and Western blot analysis. Our results showed that LIQ (0.1 mM) significantly inhibited NF-κB activation (Figure 5A). In addition, at this concentration, LIQ also significantly inhibited the RANKL-stimulated degradation of IκB-α at 10–30 min, especially at 20 min, indicating that the NF-κB pathway mediates the inhibitory effects of LIQ on osteoclast formation (Figures 5B, C). As illustrated in Figures 5D, E, LIQ predominantly suppressed JNK and p38 phosphorylation from 10 to 60 min, with the most pronounced suppression observed early at 10–30 min after LIQ treatment. The data collectively and convincingly illustrate that LIQ effectively suppresses NF-κB activity and inhibits MAPK phosphorylation upon RANKL activation.

### Suppression of NFATc1 activity and downstream factors expression by LIQ

**FIGURE 6:**
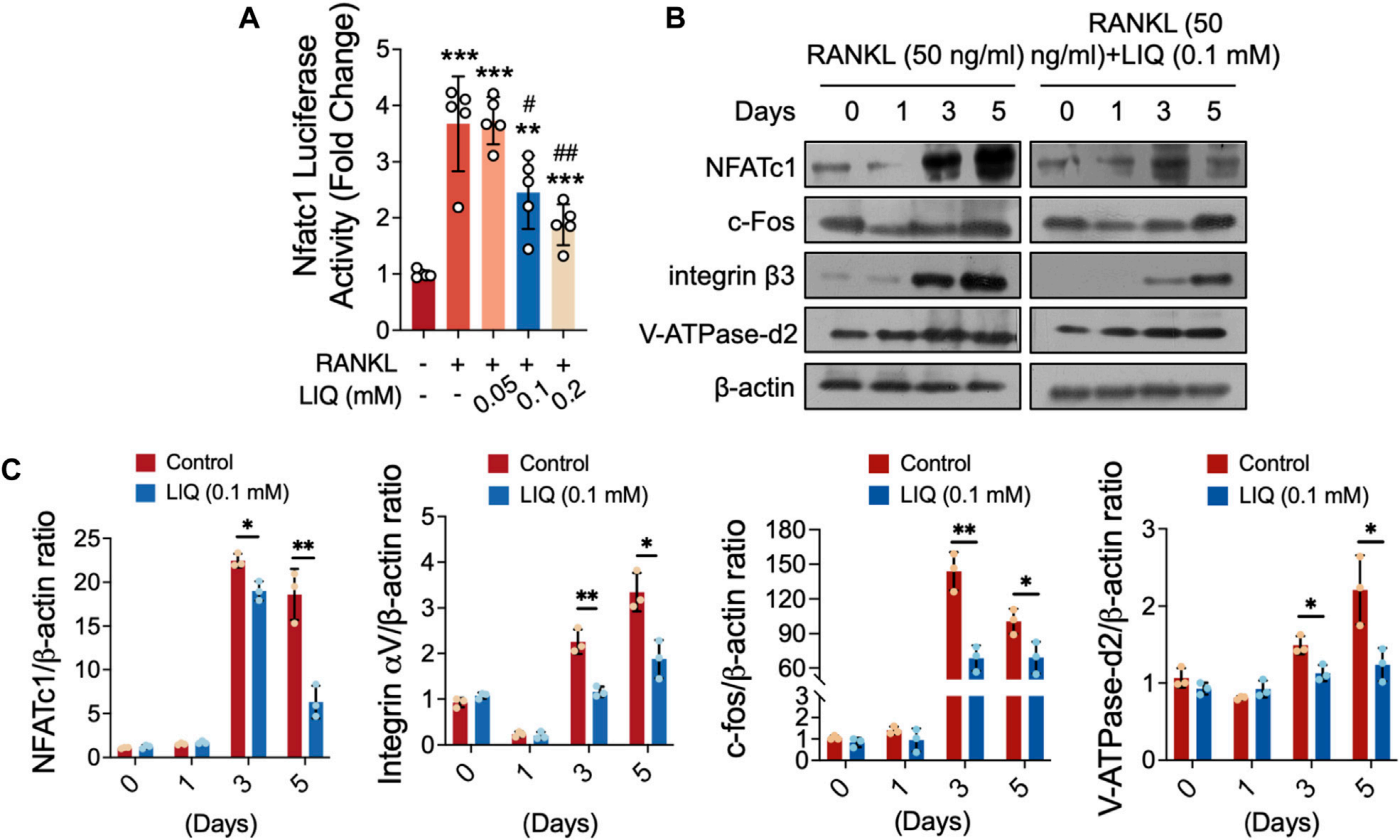
LIQ obliterates RANKL-induced nuclear translocation of Nfatc1 and modulates its protein expression. **(A)** Luciferase activity was assessed in Nfatc1-transfected RAW264.7 cells stimulated with rm-sRANKL (50 ng/mL) in the presence of various concentrations of LIQ, using an Nfatc1 luciferase construct. (**p-value <0.01, ***p-value <0.001, *versus* blank control. #*p*-value <0.05, ##*p*-value <0.01, *versus* RANKL-treated control). **(B)** Protein lysates were derived from BMM-induced osteoclasts pre-treated with LIQ (0.1 mM) and subsequently stimulated with rm-sRANKL at different time intervals. Western blot analysis was conducted employing specific antibodies targeting NFATc1, V-ATPase-d2, c-fos, and β-actin. **(C)** The relative protein levels were determined by calculating the ratio of NFATc1 to β-actin, V-ATPase-d2 to β-actin, and c-fos to β-actin using ImageJ software. (*p < 0.05, **p < 0.01, ***p < 0.01 compared to RANKL-treated and LIQ-untreated controls).

To gain a detailed understanding of the effects of LIQ on osteoclast differentiation and function, we utilized luciferase reporter gene profiling and Western blotting as analytical techniques. As shown in Figure 6A, it was confirmed by the luciferase reporter gene assay that LIQ decreased the RANKL-induced transcription of NFATc1 in a dose-dependent manner. In addition, RANKL induced the protein expression of NFATc1, which was attenuated by LIQ (Figure 6B). Significantly, after 3–5 days of administering LIQ, there was a noticeable reduction in the expression levels of essential downstream factors involved in osteoclastic bone-resorbing activity, such as VFATPase-d2, integral α V, and c-fos (Figure 6C). These discoveries underscore the substantial inhibitory impact of LIQ on the expression of pivotal genes and proteins, alongside the modulation of vital transcriptional regulators (NFATc1 and c-Fos), which are integral components of the intricate process of osteoclast differentiation.

### LIQ can prevent OVX-induced osteoporosis

**FIGURE 7:**
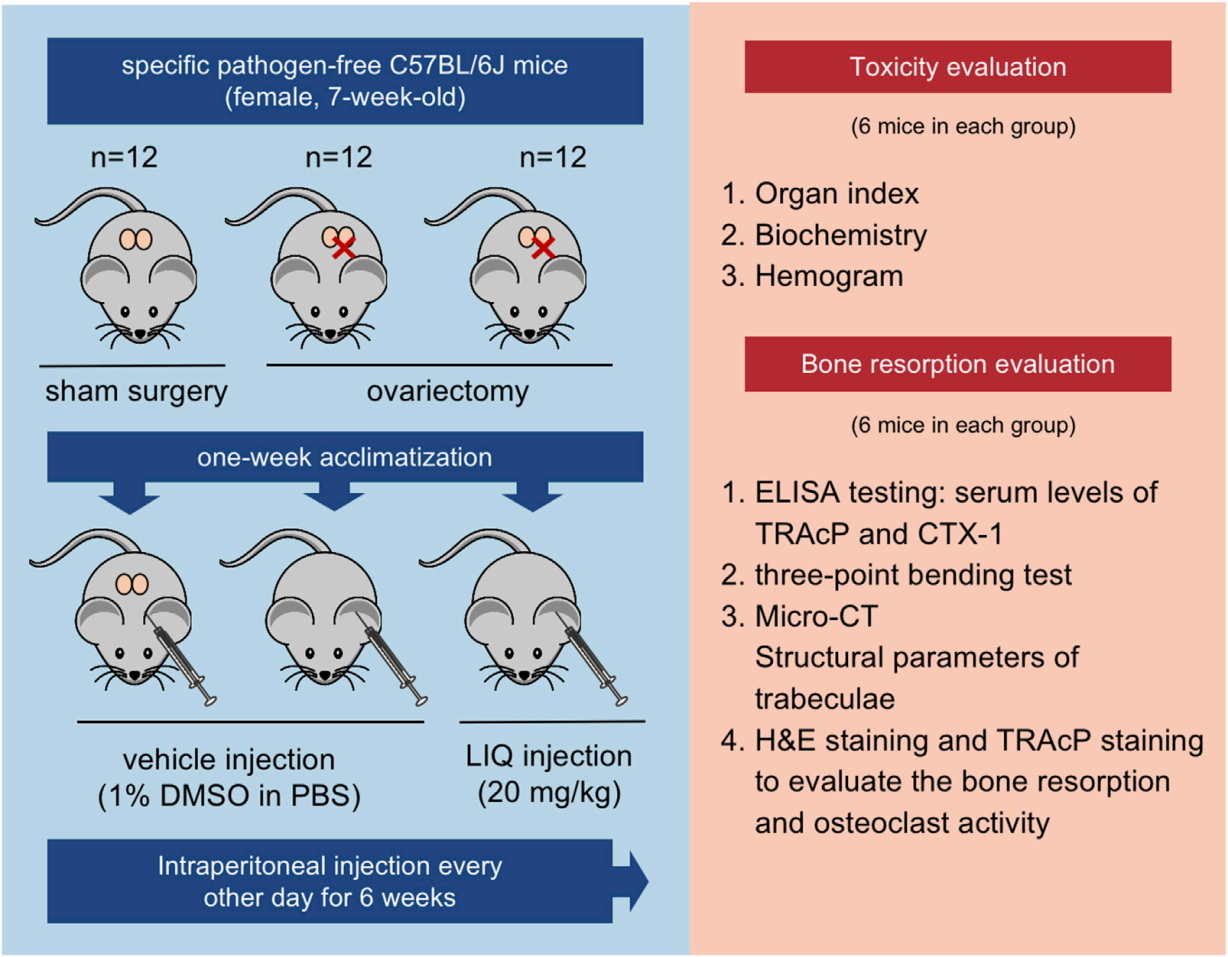
Illustration of the *in vivo* experimental protocol delineating the assessment of LIQ’s therapeutic efficacy.

We observed the effects of anti-osteoporotic agents on osteoporosis/bone loss/osteoporotic bone loss in OVX mice with postmenopausal osteoporosis (Figure 7). The mice were divided into the following groups: the sham operation (control) group, the OVX group, and the OVX + LIQ (20 mg/kg) group. Crucially, throughout the process of OVX and the intraperitoneal injection of LIQ, there were no reports of severe adverse events or mortalities. Moreover, LIQ did not induce any noticeable effects on mouse body weight (Supplementary Figure S3), laboratory biochemistry (Supplementary Table S3), or hematological profiles (Supplementary Table S4). Detailed examination of vital organs, including the lungs, liver, kidneys, heart, and spleen, revealed no significant changes in size or surface characteristics among the treatment groups (Supplementary Figure S4).

**FIGURE 8:**
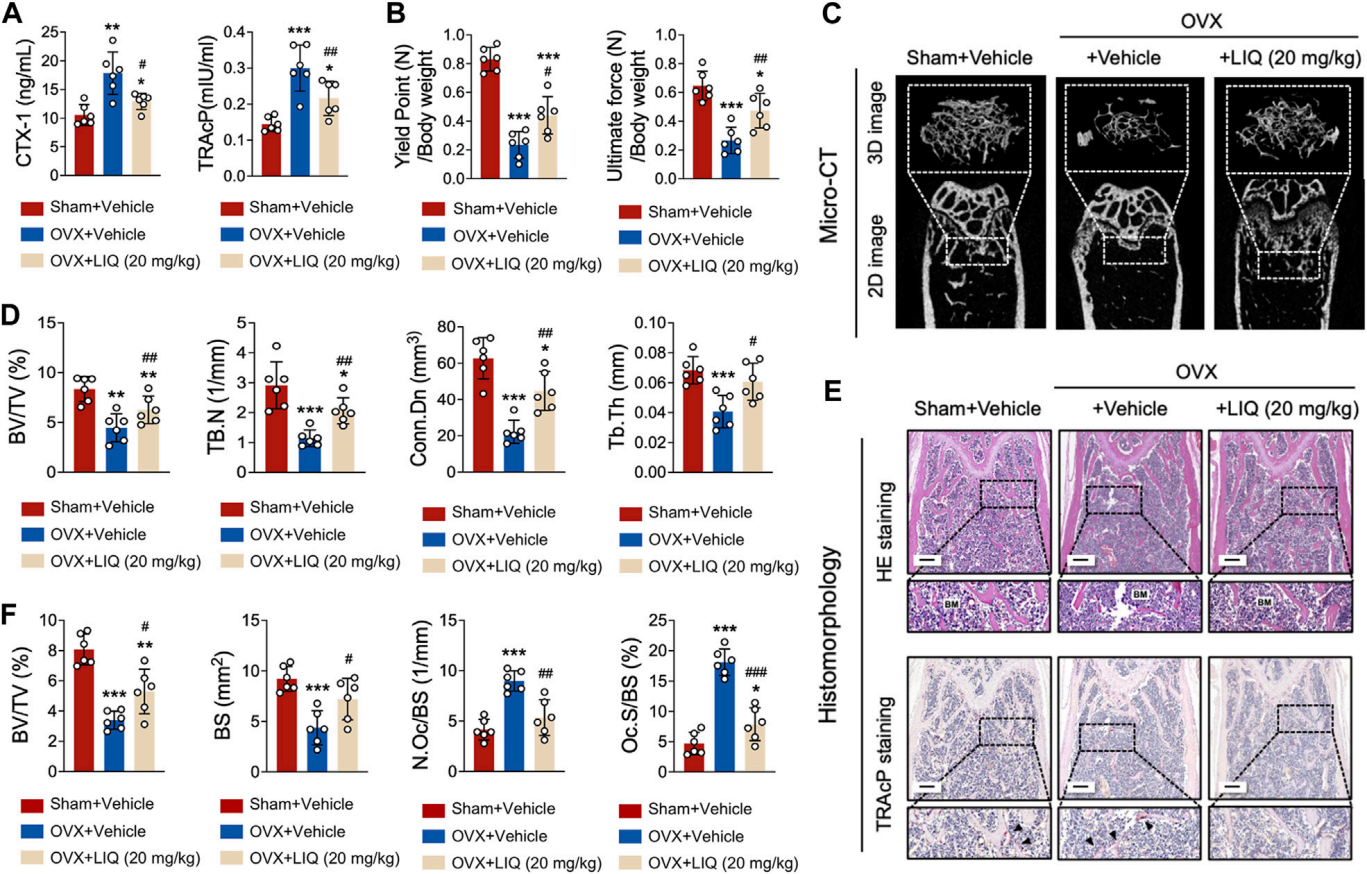
LIQ attenuates bone resorption induced by estrogen deficiency following ovariectomy. **(A)** Quantitative data presenting serum levels of TRAcP and CTX-1 in sham-operated mice, OVX mice, and the LIQ-treated group (20 mg/kg) (*n* = 6). **(B)** Quantitative measurement of ultimate force (N) and yield point (N), normalized by body weight in a cohort of mice (n = 6). **(C)** Depicting a representative three-dimensional reconstruction image and Micro-CT analysis of trabecular bone microarchitecture in the femur of each group. Results emphasize the potential protective impact of LIQ against ovariectomy-induced osteoporosis. **(D)** Quantitative analysis of BV/TV, Tb.N, Tb.Th, and Tb. Sp. **(E)** Displaying representative images of decalcified bone stained with H&E and TRAcP in each group. Magnification: ×4, scale bar: 500 μm. **(F)** Quantitative analysis of BV/TV, N. Oc/BS, and N. Ob/BS. (*p-value <0.05, **p-value <0.01, ***p-value <0.001, *versus* sham control. #*p*-value <0.05, ##*p*-value <0.01, ###*p*-value <0.001, *versus* OVX-treated control).

Upon comparative analysis with the sham-operated group, a remarkable observation emerged: the administration of LIQ resulted in a significant decrease in serum levels of TRAcP and CTX-1 in the OVX animal model. This finding highlights the potent capability of LIQ to effectively inhibit bone resorption (Figure 8A). Conducting a three-point bending analysis on the tibias extracted from each mice group revealed significant improvements in both the yield point, representing the mechanical force required to induce complete damage to the bone matrix, and the ultimate force, indicative of the bone’s overall structural integrity (Figure 8B). The three-dimensional images and bone parameters of LIQ-treated mice measured by Micro-CT showed that the bone mass increased significantly. As shown in Figures 8C, D, BV/TV and TB of OVX mice treated with LIQ increased in a dose-dependent manner, while Tb. Sep. decreased. The results showed that LIQ had a dose-dependent inhibitory effect on OVX-induced bone loss. The histological results further confirmed the protective effects of LIQ on osteoporotic bone loss. It can be seen from Figures 8E, F that BV/TV of the OVX + LIQ group is significantly higher than that of the OVX group. In addition, compared with the OVX group, N. Oc./BS and Oc. S/BS of the OVX + LIQ group were significantly reduced, indicating that LIQ could prevent osteoporotic bone loss by reducing osteoclast activity. Collectively, these findings provide compelling evidence that LIQ holds immense promise in mitigating systemic bone loss in ovariectomized animal models.

## Discussion

Currently, U.S. Food and Drug Administration-approved drugs for osteoporosis treatment included Bisphosphonates (Fuggle et al., 2022), Calcitonin (Li N. et al., 2021), and Denosumab (a human monoclonal antibody) (Kendler et al., 2022). Nevertheless, the clinical effectiveness of these medications remains unsatisfactory (Muñoz et al., 2020). For example, Bisphosphonates, which are often chosen as the first-class medicine, usually lead to low-risk atypical fractures, osteonecrosis of the jaw, and atrial fibrillation (Yuan et al., 2019). Therefore, there is a greatly urgent need for new drugs to improve the risk–benefit curve of the treatment of osteoporosis and other osteolytic diseases.

Compared with anti-osteolytic therapies mentioned above, natural compounds have several advantages, including lower cancer risk and fewer gastrointestinal diseases (Martiniakova et al., 2020). In this study, we demonstrated that LIQ, a natural compound derived from the root of *G. glabra*, can inhibit osteoclast formation and bone absorption activity by blocking RANKL-induced NF-κB activation, inhibiting osteoclast marker gene expression, reducing ROS levels, and ultimately reducing NFATc1 activity in cells. It suggests that LIQ can be used as a therapy to prevent bone destruction in osteoporosis.

First, in order to evaluate the biological function of LIQ, osteoclast differentiation and cellular activity were measured. Our findings revealed that LIQ significantly inhibited the differentiation of osteoclasts in a dose-dependent manner. The hydroxyapatite absorption test showed that LIQ inhibited the absorption by osteoclasts, indicating that LIQ influenced the absorption of mature osteoclasts.

The NF-κB/MAPK pathway, a critical signal pathway regulating osteoclast production and bone resorption, is a therapeutic target for osteolysis (Boyce et al., 2015). The initiation of the NF-κB pathway is predominantly reliant on the phosphorylation of IκB-α. Mice with NF-κB knockout demonstrate pronounced osteosclerosis owing to the absence of osteoclast formation (Xu et al., 2009). Through the NF-κB pathway, RANKL can prompt DNA binding of the c-Rel, NF-κB1 (p50), and RelA (p65) NF-κB complex (Yao et al., 2021). In addition, the inactivation of IKK-α or IKK-β is enough to inhibit osteoclast formation (Sun, 2011; Jimi et al., 2019). In Western blot and Luciferase analysis, we found that LIQ can reduce the RANKL-induced activation of NF-κB, and phosphorylation of p38 and Jnk. These data suggest that the inhibitory effects of LIQ on osteoclast formation may be related to the downregulation of the RANKL-induced NF-κB/MAPK signaling pathway.

NFATc1 serves as an indispensable transcription factor implicated in the osteoclastogenesis of BMMs. The interaction between RANKL and RANK signifies the intimate association between NF-κB and NFATc1 (Yao et al., 2021). Our findings have demonstrated a substantial reduction in both the genetic and protein expressions, as well as the activities, of NFATc1 following treatment with LIQ. Within the context of RANKL-triggered osteoclast formation, NF-κB assumes the role of an instigator for NFATc1 (Asagiri and Takayanagi, 2007; Yao et al., 2021). Past studies have elucidated that the application of dehydroxymethylepoxyquinomicin ((−)-DHMEQ), an NF-κB inhibitor, notably inhibits the activity of NFATc1 (Ma et al., 2021). Additionally, RANKL-induced Ca^2+^ oscillations mediate the activation and self-amplification of NFATc1, thus indicating the regulation of NFATc1 through the Ca^2+^–calcineurin pathway (Kang et al., 2020; Park et al., 2020). In conjunction with our observations regarding Ca^2+^ oscillations, LIQ impedes NFATc1, thereby impeding osteoclastogenesis. Consequently, LIQ exerts a dual function by obstructing both the NF-κB and Calcium–Calcineurin–NFATc1 signaling pathway.

ROS have been documented to impact the activation of NF-κB through their interference with IκB-α phosphorylation, while NF-κB, in turn, can modulate ROS activity by promoting the production of antioxidant enzymes (Morgan and Liu, 2011). Further investigations into this mechanism have revealed that LIQ, in addition to its effects on osteoclast activity, can also reduce ROS levels. Moreover, RANKL-induced signaling cascades, such as NOX1, TRAF6, and Rac1, are triggered while the production of antioxidant enzymes is concurrently diminished, leading to increased intracellular exposure to ROS and subsequent osteoclastogenesis (Xu et al., 2016; Tao et al., 2020). LIQ mitigates the accumulation of ROS in osteoclasts by suppressing the expression of TRAF6 and NOX1, thereby reducing GTP-Rac1 levels. Furthermore, LIQ promotes the levels of antioxidant enzymes, such as HO-1 (Kozakowska et al., 2014), NADPH-dependent GSR (Aquilano et al., 2014), and CAT (Kanzaki et al., 2016). Consequently, our data strongly indicate that LIQ not only limits ROS buildup but also enhances ROS scavenging, thereby naturally inhibiting the formation and activity of osteoclasts.

In order to delve deeper into the impact of LIQ on osteoporosis in an *in vivo* setting, we employed a murine model of osteoporosis induced by OVX. Through multiple testings by bone and blood samples, our findings revealed that oral administration of LIQ exerted a significant inhibitory effect on bone loss in OVX mice. Moreover, in the OVX + LIQ group, the number of osteoclasts displaying activation in the vicinity of trabeculae was notably reduced compared to the OVX group. In this study, our primary aim was to evaluate the isolated effects of Liquiritin in countering ovariectomy-induced osteoporosis, intentionally opting not to include a positive drug as a control; doing so could potentially introduce additional variables, confounding the results by masking the true effects of LIQ.

Consequently, LIQ could potentially be developed into a treatment for osteoporosis, aiming to inhibit excessive bone resorption, preserve bone density, and mitigate fracture risk in postmenopausal women and individuals at risk of osteoporosis. For individuals who cannot tolerate or have contraindications to current osteoporosis medications, LIQ could potentially serve as an alternative treatment. Its demonstrated safety profile and potential to mitigate bone loss offer an alternative option for patients with specific medical conditions or intolerances.

In the future research, conducting well-designed clinical trials to evaluate the efficacy, safety, and optimal dosage of LIQ in humans is essential. Randomized controlled trials can provide insights into LIQ’s effectiveness in preventing bone loss, improving bone density, and reducing fracture risk in individuals with osteoporosis. Investigating the long-term safety of LIQ, its effects on bone quality, and potential interactions with other medications are also crucial for its potential clinical use.

**FIGURE 9:**
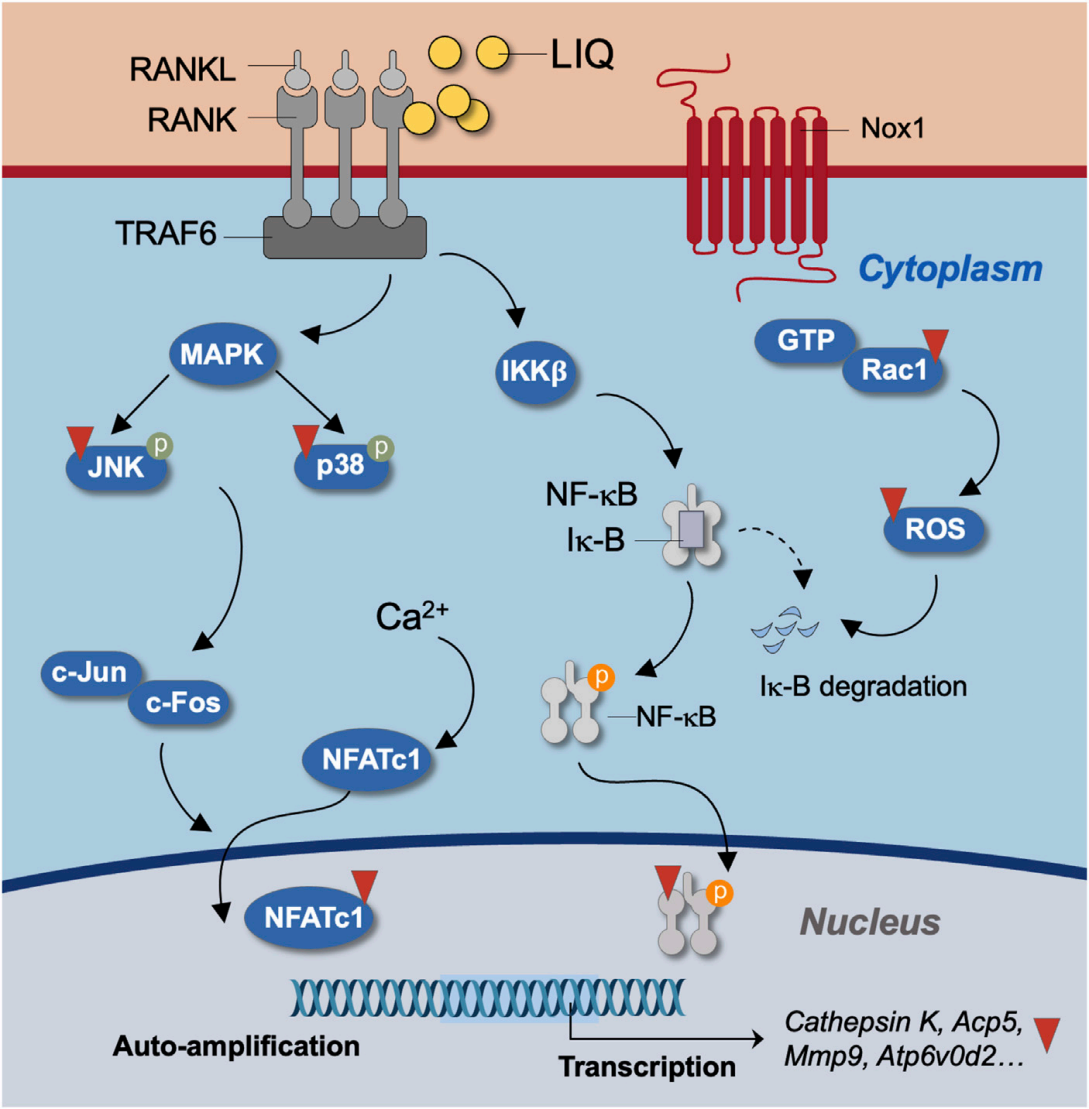
Unveiling the molecular mechanism of LIQ during osteoclastogenesis. Illustrated is a schematic diagram outlining the molecular intricacies of LIQ’s action in osteoclastogenesis. Through its inhibition of the RANKL-RANK interaction, facilitated by LIQ, a cascade of effects unfolds. This includes the suppression of MAPK and NF-κB signaling pathways, along with the modulation of calcium oscillation. These combined actions result in the curbing of NFATc1 nucleus translocation and the quelling of ROS generation.

In conclusion, our study provides compelling evidence that LIQ, a natural compound, effectively impedes the formation of osteoclasts induced by RANKL. This inhibition is achieved through the suppression of intracellular Ca^2+^ signaling and the reduction of ROS levels. This inhibition is achieved through the suppression of intracellular Ca^2+^ signaling and the reduction of ROS levels. These actions result in diminished activity of the MAPK and NF-κB signaling pathways, as well as decreased expression of NFATc1 and other genes specific to osteoclasts (Figure 9). The implications of these findings suggest that LIQ holds promise as a novel anti-resorptive drug for the treatment of osteoporosis.

## Data Availability

The raw data supporting the conclusion of this article will be made available by the authors, without undue reservation.

## Supplementary material

SUPPLEMENTARY FIGURE S1 Structural and empirical Insights into LIQ. A depiction of the structure and empirical formula of LIQ, gleaned from data sourced from PubChem (https://pubchem.ncbi.nlm.nih.gov).

SUPPLEMENTARY FIGURE S2 Monitoring body-weight changes in mice. Quantitative analysis tracks fluctuations in mice body weight within each experimental group over the course of LIQ treatment.

SUPPLEMENTARY FIGURE S3 Visualization of mice organs in experimental groups. Displayed are representative images of mice organs from respective experimental cohorts.

## Abbreviations

LIQ, Liquiritin; NFATc1, nuclear factor of activated T cells 1; MAPK, mitogen-activated protein kinase; OVX, ovariectomized; BMMs, bone marrow macrophages; NF-κB, nuclear factor nuclear factor kappa-light-chain-enhancer of activated B cells; RANKL, nuclear factor-κB ligand; ROS, reactive oxygen species; M-CSF, macrophage colony stimulating factor; TRAF6, TNF receptor-associated factor 6; NOX1, nicotinamide adenine dinucleotide phosphate oxidase 1; HO-1, heme oxygenase-1; PFA, paraformaldehyde; *c-fos*, proto-oncogene C-Fos; *Mmp9*, matrix metallopeptidase 9; *Atp6v0d2*, ATPase H^+^ transporting V0 subunit D2; *Acp5*, acid phosphatase 5, tartrate resistant; Rac1, Ras-related C3 botulinum toxin substrate 1; CAT, catalase; GTP, guanosine-5′-triphosphate; BV/TV, bone volume per tissue volume; Tb.Th, trabecular thickness; Tb. N, number of trabeculae; Conn.Dn, connectivity density; Oc.S/BS, osteoclast surface/bone surface; N.Oc/BS, number of osteoclasts/bone surface; DCF, dichlorofluorescein; ROI, region of interest.
