# Supplementary material for: A novel Glycyrrhiza glabra extract liquiritin targeting NFATc1 activity and ROS levels to counteract ovariectomy-induced osteoporosis and bone loss in murine model
Source: Front Pharmacol. 2023 Nov 8;14:1287827. doi: 10.3389/fphar.2023.1287827 (PMC10663366; doi:10.3389/fphar.2023.1287827)
Supplement: Supplementary file 1 [file Table1.DOCX]

**Table S1.** Sequences of both the forward and reverse primers of mRNAs in qRT-PCR.

| **Gene** | **Forward** | **Reverse** |
| --- | --- | --- |
| *Cathepsin K* | 5’-GGGAGAAAAACCTGAAGC-3’ | 5’ -ATTCTGGGGACTCAGAGC-3’ |
| *Acp5* | 5’-TGTGGCCATCTTTATGCT-3’ | 5’-GTCATTTCTTTGGGGCTT-3’ |
| *Atp6v0d2* | 5′-GTGAGACCTTGGAAGACCTGAA-3′ | 5′-GAGAAATGTGCTCAGGGGCT-3′ |
| *Nfatc1* | 5′-CAACGCCCTGACCACCGATAG-3′ | 5′-GGCTGCCTTCCGTCTCATAGT-3′ |
| *C-fos* | 5′-GCGAGCAACTGAGAAGAC-3′ | 5′-TTGAAACCCGAGAACATC-3′ |
| *Mmp9* | 5′-CGTGTCTG GAGATTCGACTTGA-3′ | 5′-TTGGAAACTCACACGCCAGA-3′ |
| *Gapdh* | 5′-ACCACAGTCCATGCCATCAC-3′ | 5′-TCCACCACCCTGTTGCTGTA-3′ |

**Table S2.** Antibody List.

| **Epitope/Antigen or Product name** | **Source** | **Catalog #** | **Dilution** | **Application specific details** |
| --- | --- | --- | --- | --- |
| TRAF6 | Abcam (Cambridge, MA) | ab33915 | 1: 1000 | 5% skim milk for 1 hour, overnight, 4 °C |
| NOX1 | Abcam (Cambridge, MA) | ab131088 | 1: 2000 | 5% skim milk for 1 hour, overnight, 4 °C |
| HO-1 | Abcam (Cambridge, MA) | ab68477 | 1: 1000 | 5% skim milk for 1 hour, overnight, 4 °C |
| Catalase | Abcam (Cambridge, MA) | ab76110 | 1: 1000 | 5% skim milk for 1 hour, overnight, 4 °C |
| GSR | Abcam (Cambridge, MA) | ab124995 | 1: 1000 | 5% skim milk for 1 hour, overnight, 4 °C |
| Integrin αV | Abcam (Cambridge, MA) | ab179475 | 1: 1000 | 5% skim milk for 1 hour, overnight, 4 °C |
| Nfatc1 | Abcam (Cambridge, MA) | ab25916 | 1: 1000 | 5% skim milk for 1 hour, overnight, 4 °C/2% bovine serum albumin for 1 hour, overnight, 4 °C |
| Cathepsin K | Abcam (Cambridge, MA) | ab19027 | 1: 1000 | 5% skim milk for 1 hour, overnight, 4 °C |
| IkB-$\alpha$ | Abcam (Cambridge, MA) | ab32518 | 1: 1000 | 5% skim milk for 1 hour, overnight, 4 °C |
| p-p38 | Cell Signaling Technology (Beverly, MA) | 4511T | 1: 3000 | 5% skim milk for 1 hour, overnight, 4 °C |
| p38 | Cell Signaling Technology (Beverly, MA) | 8690T | 1: 1000 | 5% skim milk for 1 hour, overnight, 4 °C |
| p-Jnk | Cell Signaling Technology (Beverly, MA) | 9255s | 1: 2000 | 5% skim milk for 1 hour, overnight, 4 °C |
| Jnk | Cell Signaling Technology (Beverly, MA) | 9252T | 1: 1000 | 5% skim milk for 1 hour, overnight, 4 °C |
| $\beta$-actin | Beijing Biodragon Immunotechnologies (Beijing, China) | B1033 | 1: 3000 | 5% skim milk for 1 hour, overnight, 4 °C |

**Table S3.** Analysis of clinical biochemistry of mouse blood.

| **Items** | **Sham group (n=6)** | **OVX group (n=6)** | **OVX+PIN group (n=6)** |
| --- | --- | --- | --- |
| ALB (g/dL) | 2.28$\pm$0.40 | 2.89$\pm$0.07 | 2.45$\pm$0.40 |
| ALT (U/L) | 32.06$\pm3$.21 | 35.15$\pm$1.22 | 36.18$\pm3$.13 |
| AST (U/L) | 96.12$\pm$2.46 | 97.81$\pm$3.18 | 96.13$\pm$4.36 |
| TBIL (mg/dL) | 0.28$\pm$0.04 | 0.23$\pm$0.04 | 0.27$\pm$0.03 |
| BUN (mg/dL) | 16.09$\pm$5.43 | 17.25$\pm$3.17 | 17.23$\pm$1.28 |
| CHOL (mg/dL) | 97.16$\pm$8.21 | 93.24$\pm$6.14 | 98.32$\pm$4.26 |
| CRE (mg/dL) | 0.25$\pm$0.03 | 0.27$\pm$0.02 | 0.24$\pm$0.03 |
| GGT (U/L) | 6.18$\pm$0.06 | 6.27$\pm$0.07 | 6.27$\pm$0.04 |
| GLU (mg/dL) | 224.03$\pm$15.28 | 242.19$\pm$13.89 | 271.76$\pm$18.33 |

*ALB, albumin; ALT, alanine transaminase; AST, aspartate transaminase; TBIL, total bilirubin level; BUN, blood urea nitrogen; CHOL, cholesterol; CRE, creatinine; GGT, gamma glutamyl transpeptidase; GLU, glucose.

**Table S4.** Hematology in the blood of mice.

| **Items** | **Sham group (n=6)** | **OVX group (n=6)** | **OVX+PIN group (n=6)** |
| --- | --- | --- | --- |
| WBC (K/$\mu$L) | 6.28$\pm$0.25 | 5.22$\pm$0.17 | 5.02$\pm$0.39 |
| RBC (M/$\mu$L) | 8.36$\pm$1.19 | 8.45$\pm$1.22 | 9.12$\pm$2.03 |
| HGB (g/dL) | 14.77$\pm$2.29 | 13.16$\pm1$.27 | 14.65$\pm$1.22 |
| HCT (%) | 51.236$\pm$2.94 | 46.53$\pm$2.36 | 50.17$\pm$7.18 |
| MCV (fL) | 44.35$\pm$5.35 | 50.33$\pm$6.27 | 49.21$\pm$8.84 |
| MCH (pg) | 16.75$\pm$2.39 | 18.01$\pm$1.24 | 17.88$\pm$3.44 |
| MCHC (g/dL) | 24.87$\pm$6.35 | 25.72$\pm$5.22 | 27.19$\pm$3.28 |
| PLT (K/$\mu$L) | 1377.27$\pm$67.29 | 1318.28$\pm$61.29 | 1318.92$\pm$90.22 |
| RDW (%) | 18.22$\pm$2.10 | 19.23$\pm$5.24 | 18.32$\pm$4.19 |
| MPV (fL) | 4.02$\pm$0.41 | 3.28$\pm$0.28 | 4.03$\pm$0.28 |
| NEUT (K/$\mu$L) | 1.72$\pm$0.03 | 1.86$\pm$0.04 | 1.92$\pm$0.03 |
| LYMPH (k/$\mu$L) | 8.11$\pm$0.41 | 8.10$\pm$0.17 | 7.52$\pm$0.55 |
| MONO (k/$\mu$L) | 0.63$\pm$0.03 | 0.62$\pm$0.04 | 0.58$\pm$0.03 |
| EO (k/$\mu$L) | 0.20$\pm$0.04 | 0.26$\pm$0.02 | 0.19$\pm$0.02 |
| BASO (mg/dL) | 0.06$\pm$0.00 | 0.08$\pm$0.01 | 0.06$\pm$0.00 |

*WBS, white blood cell count; RBC, red blood cell count; HGB, hemoglobin; HCT, hematocrit; MCV, mean corpuscular volume; MCH, mean corpuscular hemoglobin; MCHC, mean corpuscular hemoglobin concentration; PLT, platelet; RDW, red blood cell volume distribution width; PDW, platelet distribution width; MPV, mean platelet volume; NEUT, neutrophil count; LYMPH, lymphocyte count; MONO, monocyte count; EO, eosinophil count; BASO, basophil count.
