# Supplementary figures and images for: A novel Glycyrrhiza glabra extract liquiritin targeting NFATc1 activity and ROS levels to counteract ovariectomy-induced osteoporosis and bone loss in murine model
Source: Front Pharmacol. 2023 Nov 8;14:1287827. doi: 10.3389/fphar.2023.1287827 (PMC10663366; doi:10.3389/fphar.2023.1287827)

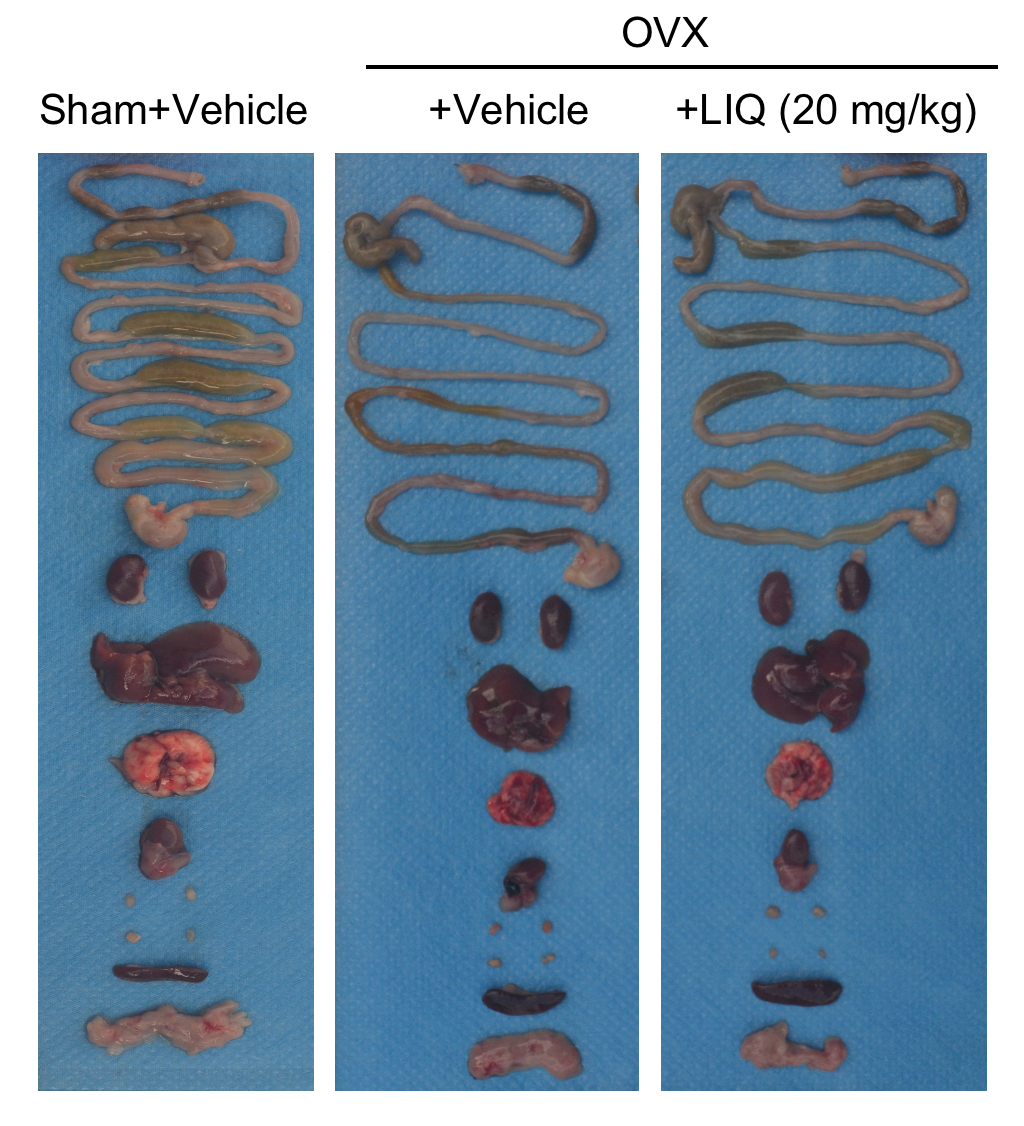

Supplement: Supplementary file 2 [file Image3.TIF]

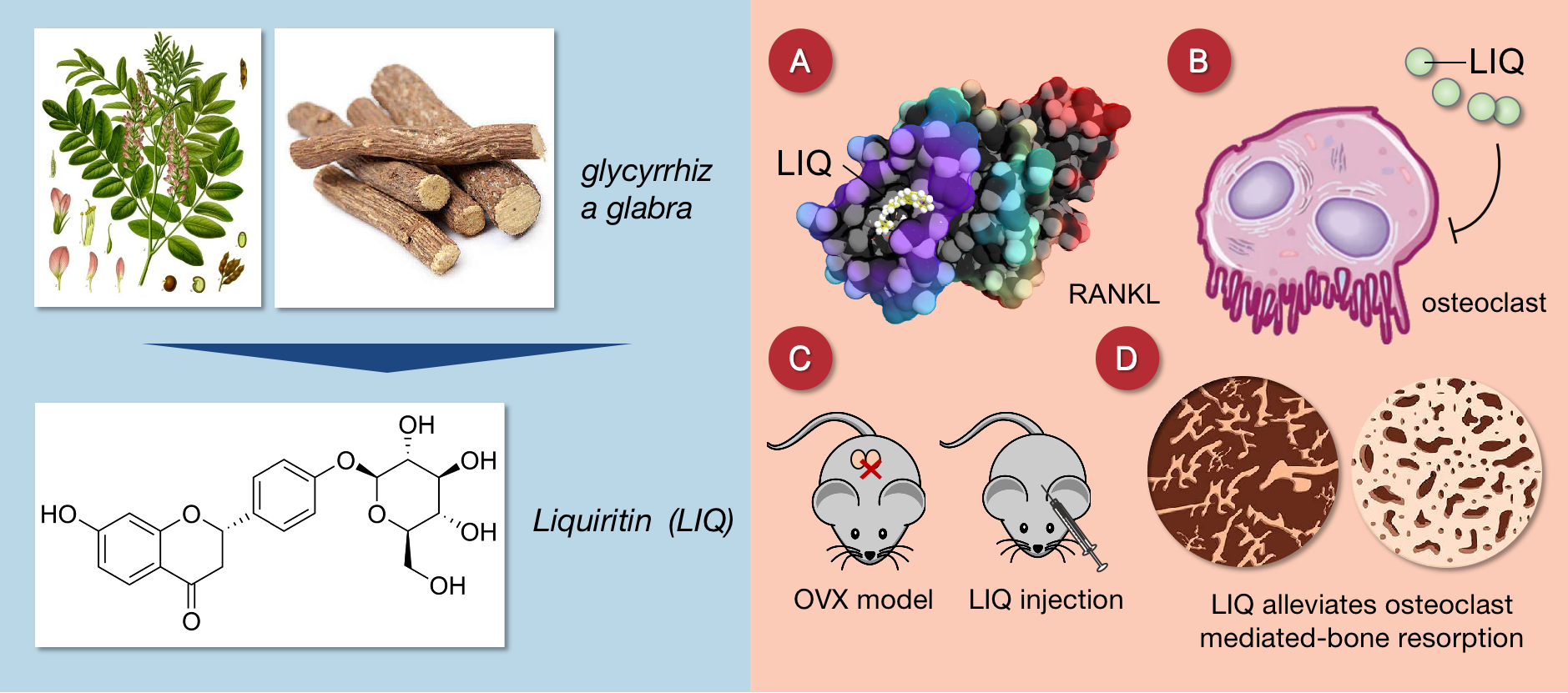

Supplement: Supplementary file 3 [file Image4.TIF]

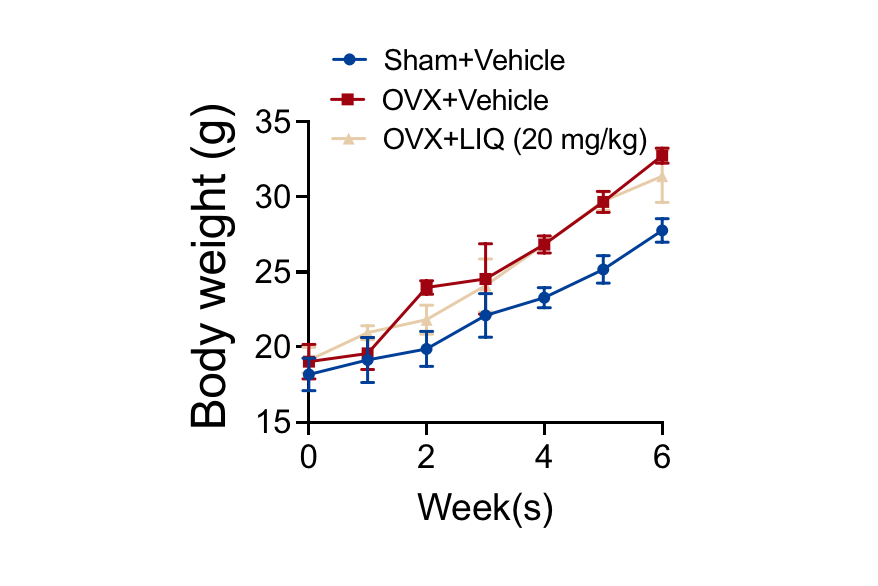

Supplement: Supplementary file 4 [file Image2.TIF]

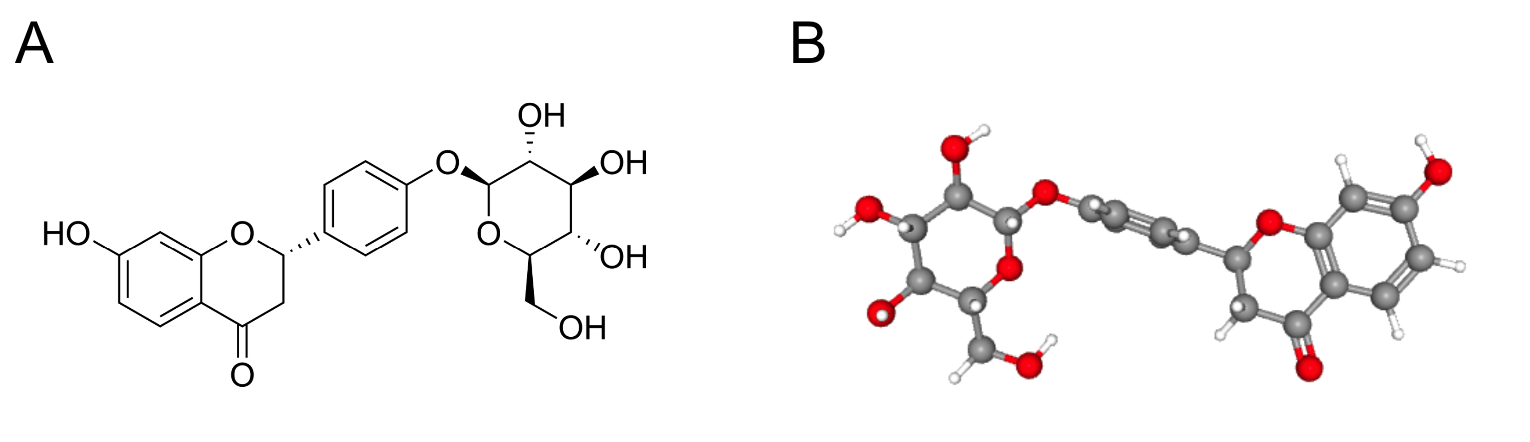

Supplement: Supplementary file 5 [file Image1.TIF]
